# The role of coccolithophore calcification in bioengineering their environment

**DOI:** 10.1098/rspb.2016.1099

**Published:** 2016-06-29

**Authors:** Kevin J. Flynn, Darren R. Clark, Glen Wheeler

**Affiliations:** 1 College of Science, Swansea University, Swansea SA2 8PP, UK; 2 Plymouth Marine Laboratory, Prospect Place, Plymouth PL1 3DH, UK; 3 Marine Biological Association, Citadel Hill, Plymouth, PL1 2PB, UK

**Keywords:** ocean acidification, *Emiliania*, coccolithophorid, coccolith, climate change, bioengineering

## Abstract

Coccolithophorids are enigmatic plankton that produce calcium carbonate coccoliths, which over geological time have buried atmospheric CO_2_ into limestone, changing both the atmosphere and geology of the Earth. However, the role of coccoliths for the proliferation of these organisms remains unclear; suggestions include roles in anti-predation, enhanced photosynthesis and sun-screening. Here we test the hypothesis that calcification stabilizes the pH of the seawater proximate to the organisms, providing a level of acidification countering the detrimental basification that occurs during net photosynthesis. Such bioengineering provides a more stable pH environment for growth and fits the empirical evidence for changes in rates of calcification under different environmental conditions. Under this scenario, simulations suggest that the optimal production ratio of inorganic to organic particulate C (PIC : POC_prod_) will be lower (by approx. 20%) with ocean acidification and that overproduction of coccoliths in a future acidified ocean, where pH buffering is weaker, presents a risk to calcifying cells.

## 1. Introduction

Biogenically deposited limestone is a feature of the geology of the Earth, providing a reservoir for C that formally existed as atmospheric CO_2_ [1]. Much limestone originates from the activity of coccolithophids, a cosmopolitan and ecologically important group of phytoplankton [2,3], characterized by their ability to synthesize coccoliths of calcium carbonate (CaCO_3_). Coccolith production is a major contributor to global biogeochemical cycles, enhancing C-export rates to the deep ocean by providing ballast to marine particles incorporating coccolithophorids [2,4]. Despite this significance, the functions of coccolith production for the organisms themselves remain unclear; suggestions have included protection against grazers, modulation of sinking rates and regulation of incident light [5,6]. Although roles may be species and environment specific [5–9], clear evidence supporting any of these functions are lacking [5,10]. Here we present evidence for a role of calcification in controlling, or bioengineering, the pH environment inhabited by coccolithophorids. While the driver for organism evolution is the environment of immediate relevance to those organisms (here, for pH, that would be water immediately adjacent to the cells, and over periods of days during bloom growth; [11]), the cumulative consequences of coccolithophorid activity have had major impacts over planetary and geological scales [2,4].

All organisms modify their environment, usually to their detriment by removing resources and releasing waste. In marine ecosystems, photosynthetic phytoplankton remove CO_2_ and nutrients to support their growth, thereby modifying carbonate chemistry and increasing seawater pH. This basification is deleterious to phytoplankton growth; a stable pH is better [12]. Basification accompanying CO_2_ drawdown is most pronounced at high rates of primary production. However, even at low cell densities, CO_2_ uptake can influence pH in the microenvironment around larger phytoplankton cells and aggregates of smaller cells [11]. Consequently, phytoplankton must tolerate changes in external pH resulting from physiological processes, set against the need to maintain an internal pH range favourable to growth.

Calcification occurs intracellularly in coccolithophorids and mature coccoliths are secreted, forming a coccosphere from which coccoliths may detach [13]. A crucial feature of coccolith production is that precipitation of CaCO_3_ results in H^+^ production. Consequently, a direct role in C-acquisition has been proposed, with calcification-derived H^+^ facilitating conversion of bicarbonate to CO_2_ for C-fixation [14,15]. However, modulation of calcification in the coccolithophorid *Emiliania huxleyi* by manipulation of seawater [Ca^2+^] has little effect on photosynthetic C-fixation or growth [16–18]. Calcification also does not act as a C-concentrating mechanism in *E. huxleyi* under C-limiting conditions [19]. Consequently, it appears unlikely that calcification and photosynthetic carbon uptake link directly. However, H^+^ production by calcification could benefit the cell in another way. Photosynthesis and respiration lead to an increase or decrease in proximal pH, respectively, with net photosynthesis leading to detrimental basification of bulk water at higher cell densities [12,20]. Uniquely for coccolithophorids, calcification-derived H^+^ could counter this basification, potentially maintaining a more stable pH around the cell through careful modulation of the ratio between particulate inorganic carbon (PIC) and particulate organic carbon (POC) synthesis.

We have experimental evidence that growth at a stable pH is of benefit to phytoplankton growth, including for *Emiliania* [12,21], and we have a dynamic model that explains the coupled dynamics of biology and chemistry during growth in those experiments [12]. Here we operate that model system to consider the effects that calcification has on pH and compare those results (in terms of the production ratio of particulate inorganic to organic carbon; PIC : POC_prod_) against the literature data for the cellular ratio (PIC : POC_cell_). We hypothesize that appropriate modulation between calcification and photosynthesis uniquely provides a mechanism through which coccolithophorids may bioengineer their environment, providing a stable proximal pH to optimize growth conditions for individual cells during enhanced primary production.

## 2. Material and methods

Simulations were run of the growth of a 5 µm diameter calcifying phytoplankton, nominally referred to as *Emiliania* hereafter. The model provides a variable stoichiometric (i.e. C : N : P : Chl) description of phytoplankton growth, including limitations by resource availability (CO_2_, nitrate, ammonium, phosphate, light). The maximum specific growth rate used for the default simulations was 1 d^−1^; this value is in keeping with expectations for *Emiliania* [22]. The model was run within a description of the physico-chemical environment that included a full accounting of changes in carbonate chemistry and [H^+^] with photosynthesis (including nutrient assimilation), respiration, calcification, gas exchange at the air–sea surface and also at the interface between the mixed layer photic zone and the deeper water. Growth was simulated in various water column scenarios, similar to those used before [11,12]; surface light was provided at 1000 µmol m^−2^ s^−1^ in a 12 L : 12 D cycle, and nutrients were supplied at a default 16 µM nitrate (N) and 1 µM phosphate (P).

Simulations were run under different conditions, assuming a fixed ratio of bioproduction of particulate inorganic C (coccoliths) and organic C (biomass). Hereafter, the mass ratio of these productions is referred to as PIC : POC_prod_. PIC production was simulated to occur concurrently with C-fixation. Algal bloom growth was simulated over 20 d, or for the slow growth and high nutrient simulations, 40 d; by the end the population had entered into the plateau phase of nutrient-limited growth. The initial [H^+^] was an equilibrium value with the applied atmospheric *p*CO_2_ at a salinity of 35, and at 16°C. It was assumed, in simulations starting with different initial values of *p*CO_2_, that the phytoplankton adapt to prevailing conditions, and hence that growth rates in adapted strains would be the same. This is consistent with empirical evidence [23]. No effect of variable pH upon growth rate was assumed (CF, [11]) because the objective here was to determine the value of PIC : POC_prod_ that maintained the external pH close to a constant (and thence assumed optimal) value.

Changes in seawater [H^+^] (i.e. pH) during bloom development reflect mainly physical fluxes of CO_2_ and carbonate ions together with rates of primary production and respiration. Depending on PIC : POC_prod_ exhibited by *Emiliania* this net change leads to a basification (pH rise) or an acidification (pH fall). For display purposes, the extremes of variation in H^+^ ion concentration [H^+^] over the simulated growth period have been normalized to the initial [H^+^] used for that simulation. This variation is expressed as a % δH^+^. Deviations in [H^+^] at high PIC : POC_prod_ are associated with acidification, and deviations at low PIC : POC_prod_ are associated with basification; a non-calcifying phytoplankter (i.e. PIC : POC_prod_ = 0) would generate maximum levels of basification.

Further details are given in the electronic supplementary material.

## 3. Results

Through simulations, we explored how the balance between photosynthesis and calcification rates influences the pH around coccolithophorids. We determined PIC : POC_prod_ values that most closely maintained constant pH, whatever that start pH may be, assuming (i) growth is maximized in a fixed pH environment [12] and (ii) *Emiliania* would evolve to maximize growth at the applied *p*CO_2_ [23]. Optimal PIC : POC_prod_ is thus defined as that which results in the lowest change in [H^+^] (i.e. 0 δH^+^) during simulated bloom events.

**Figure 1.**
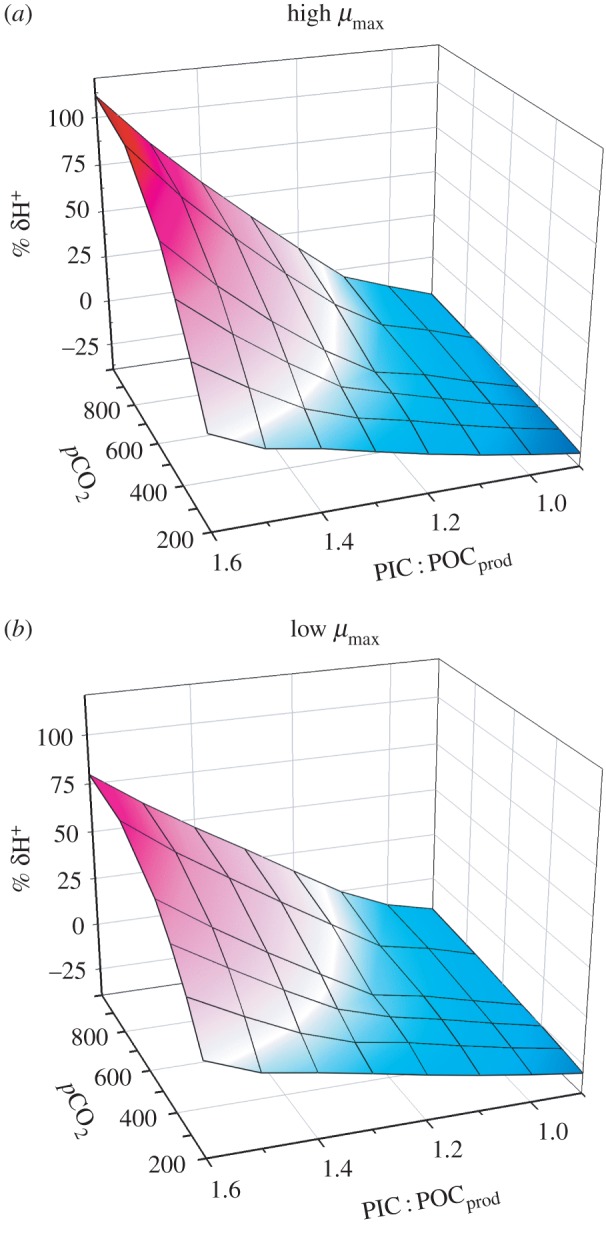
Deviation (δH^+^) from the initial [H^+^] during simulated growth of *Emiliania* under different atmospheric values of *p*CO_2_, and different PIC : POC_prod_. Assuming that growth is maximized in a stable pH environment, optimal PIC : POC_prod_ aligns with minimum δH^+^. Growth was simulated in a well-mixed water column of 25 m depth, with 16 µM nitrate, 16°C and 10 m s^−1^ surface wind speed. Positive values of δH^+^ (see the electronic supplementary material for details) show deviations at high PIC : POC_prod_ to be associated with acidification (red gradation), with negative values at low PIC : POC_prod_ associated with basification (blue gradation). White indicates zero or minimal δH^+^. (*a*) Events for the default maximum growth rate (1 d^−1^ in a 12 L : 12 D cycle) and (*b*) for a maximum growth rate of half this value. (Online version in colour.)

Figure 1 presents plots of δH^+^ against atmospheric *p*CO_2_ and PIC : POC_prod_. As *p*CO_2_ increases (i.e. from pre-industrial to future pCO_2_ scenarios), so the optimal PIC : POC_prod_ declines from 1.5 at 200 ppm *p*CO_2_ to 1.05 at 1000 ppm. Maintaining a high PIC : POC_prod_ becomes increasingly deleterious at high *p*CO_2_. The absolute deviation in [H^+^] was greatest at high *p*CO_2_ (OA conditions) as the initial pH and buffering capacity of seawater is lower; under OA, the optimal PIC : POC_prod_ is lowered significantly and selection against high PIC : POC_prod_ may be expected. The situation is similar with high and low growth rate *Emiliania* (figure 1*a* versus *b*), but because seawater pH reflects both CO_2_ consumption and atmospheric gas exchange, selective pressures to optimize PIC : POC_prod_ are expected to diminish in slower growing systems.

**Figure 2.**
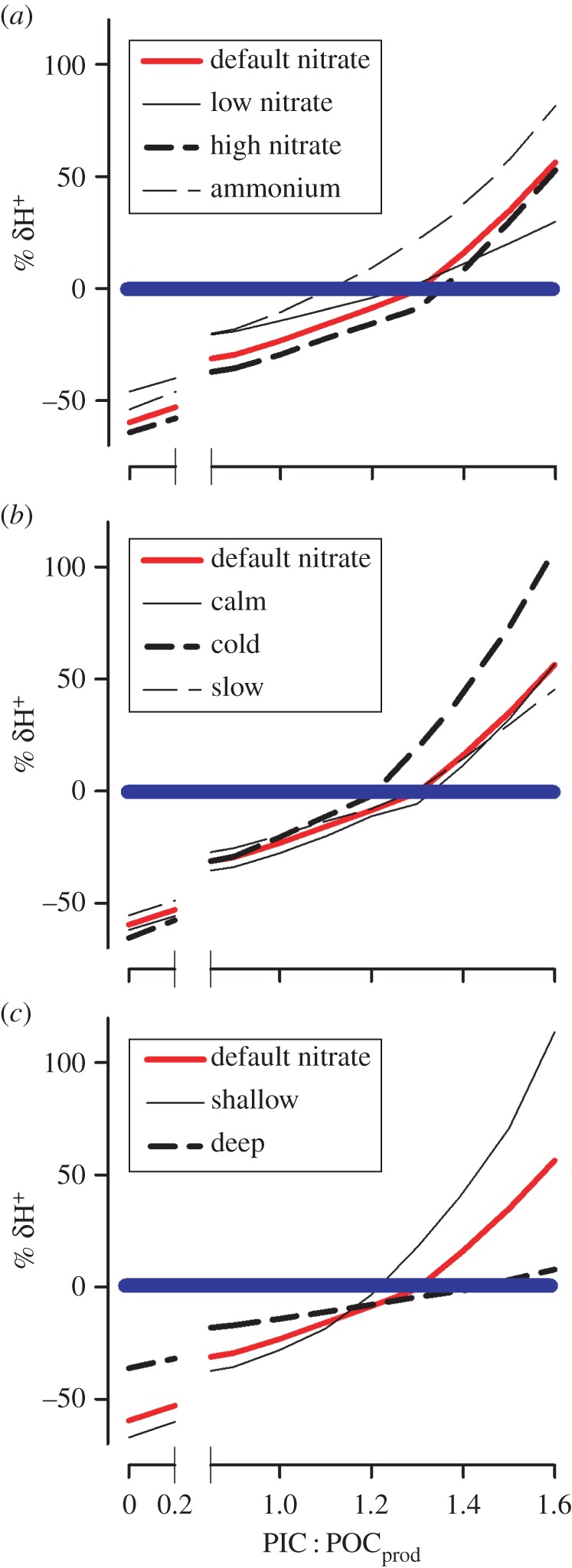
Deviation (δH^+^) from the initial [H^+^] during simulated growth of *Emiliania* under different situations at an atmospheric *p*CO_2_ of 400 ppm, and different PIC : POC_prod_. Assuming that growth is maximized in a stable pH environment, optimal PIC : POC_prod_ aligns with the minimum δH^+^ (see the electronic supplementary material for further details). The default (control) growth was simulated in a well-mixed water column of 25 m depth, with 16 µM nitrate, 16°C and 10 m s^−1^ surface wind speed, and a maximum growth rate of 1 d^−1^ in a 12 L : 12 D cycle. Positive values of δH^+^ (see the electronic supplementary material for details) show deviations at high PIC : POC_prod_ to be associated with acidification, with negative values at low PIC : POC_prod_ associated with basification. The thick blue line indicates zero δH^+^. (*a*) Events with nitrate supplied at half (low nitrate) or double (high nitrate) the default concentrations, or using ammonium rather than nitrate. (*b*) Events under calm conditions (wind spend 0 m s^−1^), cold (5°C) or with slow growth (half the default growth rate). (*c*) Events in shallow (10 m) or deep (50 m) mixed layers. (Online version in colour.)

Figure 2 presents comparative plots of simulations at 400 ppm *p*CO_2_ (extant atmosphere) with different nutrient loads or physical conditions, and include the value of δH^+^ for blooms of a phytoplankton with zero-calcification potential (i.e. PIC : POC_prod_ = 0). Higher nutrient loads drive stronger deviations in [H^+^], and slightly higher optimal PIC : POC_prod_. Growth using ammonium (which promotes acidification) drives a lower optimal PIC : POC_prod_ and higher δH^+^ at high PIC : POC_prod_ (figure 2*a*). Calm conditions, minimizing air–sea CO_2_ exchange, are conducive to greater basification during photosynthesis and drive a higher optimal PIC : POC_prod_ (figure 2*b*). Colder water conditions (with the default wind speed) are associated with changes in the carbonate chemistry and gas exchange rates that lower optimal PIC : POC_prod_. Slow growth (consistent with figure 1*b*) flattens the response curve and also slightly lowers the optimal PIC : POC_prod_ (figure 2*b*). Growth in deep mixed layers is associated with lower water column gas exchange rates, and also with lower growth rates (due to light attenuation); here the range of δH^+^ are lowered and the optimal PIC : POC_prod_ is also elevated. Shallower mixed layers not only accentuate δH^+^, lower the optimal PIC : POC_prod_, but also show very high δH^+^ when *Emiliania* grows with high PIC : POC_prod_.

## 4. Discussion

Phytoplankton growth is optimal at constant pH; basification and acidification are detrimental [12,20]. Calcification can radically alter the environmental [H^+^], potentially countering photosynthesis-driven basification and also causing acidification of proximal waters (and thence bulk water) if inadequately modulated. Sensitivity of *E. huxleyi* to decreased seawater pH is due to the increase in [H^+^], rather than changes in other carbonate system parameters [24]. This may relate to their unusual mechanisms of pH homeostasis, as H^+^ efflux via voltage-gated H^+^ channels in the plasma membrane is less effective at lower seawater pH [25,26]. Growth of coccolithophores is also inhibited at pH > 8.6 [12,24]. The energetic costs associated with calcification as a mechanism to stabilize external pH, as required through our hypothesis, versus the cost of pH homeostasis through H^+^ pumping clearly warrants investigation.

Does *Emiliania* bioengineer its external pH to optimize growth? It should be noted that PIC : POC_cell_ is expected to be lower than PIC : POC_prod_, as reported by the model, as coccoliths may detach from the cells. Comparisons between these ratios must thus refer to trends. Taking this into account, it appears to be more than coincidental that the optimal PIC : POC_prod_ indicated by our simulations aligns with experimental measurements of PIC : POC_cell_ varying with carbonate system parameters [6,14]. PIC : POC_cell_ of *E. huxleyi* decreases from 1.0 to 0.3 with increasing *p*CO_2_; a meta-analysis shows this trend is consistent across a wide range of strains and experimental conditions [7,8] and with other calcifying species [9]. At high pH > 8.5, we might expect PIC : POC_prod_ to increase further in order to drive a decrease in pH. However, PIC : POC_cell_ of *Coccolithus pelagicus* cells at pH > 8.5 is less than 1; a concomitant decrease in growth rate with elevated pH may indicate an inability to maintain high calcification rates in this species [9].

The most likely situation in which coccolithophores may act to modify their environment is during high growth at high cell density, during blooms. Globally, *E. huxleyi* is currently the most abundant bloom forming species, with natural bloom densities ranging from 1 to 30 × 10^6^ cells l^−1^ in open ocean and coastal environments [27,28]. Our default simulations describe a peak nutrient-replete bloom size of 80 × 10^6^ cells l^−1^ (see the electronic supplementary material), but in the absence of calcification (i.e. PIC : POC_prod_ = 0), significant deviations in bulk seawater pH are apparent at cell densities in line with natural blooms (20–40 × 10^6^ cells l^−1^). It thus appears quite feasible that calcification in nature offers a selective advantage by maintaining an environmental pH more favourable for growth.

Primary production at a high PIC : POC_prod_ is potentially dangerous, due to the risk of seawater acidification. Careful modulation of calcification may thus be expected, and indeed *Emiliania* (and other calcifying phytoplankton) have scope for fine regulation through internalization of the calcification process [29,30]. Close regulation of calcification is particularly important in dense suspensions; while basification is highest in such conditions and hence one may expect a high PIC : POC_prod_ to be of advantage, the collective drawdown of DIC by both photosynthesis and calcification lowers the buffering capacity of the seawater. Under such conditions, seawater pH stability becomes weakened and acidification due to increasing system respiration at the peak of a bloom could lead to a sudden collapse of the population. Starting this whole bloom growth process in acidified waters (with OA, at high *p*CO_2_) thus places a greater risk of further acidification for high calcifiers.

Irrespective of whether or not coccolithophorids such as *Emiliania* evolved calcification to bioengineer their environment to stabilize pH during growth, stabilizing the pH proximal to the cell is clearly advantageous [12,21]. Simulations for growth with zero calcification (PIC : POC_prod_ = 0; figure 2) demonstrate the scope of compensation for basification offered by calcification, and subsequently that calcification has clear potential to be advantageous in blooms essentially dominated by calcifying cells. Growth in mixed blooms, with co-occurring non-calcifying species, would still be advantageous to the coccolithophorid if the non-calcifying species were disadvantaged by growth at the fixed pH. This is possible if the non-calcifier was adapted to grow best at the raised pH more typical of conditions during the basification that accompanies normal phytoplankton growth [12].

Simulations demonstrate that under OA scenarios selection for growth of coccolithophorids with lower PIC : POC_prod_ is to be expected (as a combination of the optimal PIC : POC_prod_ for low δH^+^, and also avoiding the risk of high δH^+^ at high PIC : POC_prod_), and that optimal ratios will be lower again in cold waters (figure 2*b*), where ammonium supports growth (figure 2*a*) and also in shallow mixed layers (figure 2*c*). Together with a decrease in the optimal PIC : POC_prod_, our results suggest calcification in coccolithophorids will decline significantly in the more acidic future ocean by approximately 25% as atmospheric *p*CO_2_ exceeds 750 ppm. In large blooms, the removal of the resource for photosynthesis, through calcification, is also detrimental for pH stability. Accordingly, we can expect coccolithophorids to adapt to changes in environmental conditions impacting upon pH stability by altering their PIC : POC_prod_. For climate change models, especially over palaeological timescales, it may be possible to simulate changes in rates of calcification making reference to optimal PIC : POC_prod_. It is, however, important to note that the process of calcification on biogeochemical scales is a function of both the success of the organisms in forming large blooms (linked to nutrient loads, light, grazing and to physico-chemical oceanography) and also of the calcification of the individual organisms. The implications of the proposed bioengineering event for organisms other than the coccolithophorids is thus also of importance.

## Supplementary Material

Additional Materials and Methods

## Data accessibility

Model code is available and as described in the electronic supplementary material (and references cited therein); code may also be obtained in the form used for this work (i.e. as a Powersim Constructor file) from the corresponding author.

## References

[1] Westbroek P et al. 1993 A model system approach to biological climate forcing—the example of *Emiliania huxleyi*. Glob. Planet. Change 8, 27–46. (10.1016/0921-8181(93)90061-R)

[2] Poulton AJ, Adey TR, Balch WM, Holligan PM 2007 Relating coccolithophore calcification rates to phytoplankton community dynamics: regional differences and implications for carbon export. Deep Sea Res. II 54, 538–557. (10.1016/j.dsr2.2006.12.003)

[3] Read BA et al. 2013 Pan genome of the phytoplankton *Emiliania* underpins its global distribution. Nature 499, 209–213. (10.1038/nature12221) 23760476

[4] Rost B, Riebesell U 2004 Coccolithophores and the biological pump: responses to environmental changes. In Coccolithophores, from molecular processes to global impact (ed. Thierstein HR, Young JR), pp. 99–125. Heidelberg, Germany: Springer.

[5] Raven JA, Crawfurd K 2012 Environmental controls on coccolithophore calcification. Mar. Ecol. Prog. Ser. 470, 137–166. (10.3354/meps09993)

[6] Müller MN, Trull TW, Hallegraeff GM 2015 Differing responses of three Southern Ocean *Emiliania huxleyi* ecotypes to changing seawater carbonate chemistry. Mar. Ecol. Prog. Ser. 531, 81–90. (10.3354/meps11309)

[7] Findlay HS, Calosi P, Crawfurd K 2011 Determinants of the PIC:POC response in the coccolithophorid *Emiliania huxleyi* under future ocean acidification scenarios. Limnol. Oceanogr. 56, 1168–1178. (10.4319/lo.2011.56.3.1168)

[8] Meyer J, Riebesell U 2015 Reviews and syntheses: responses of coccolithophores to ocean acidification: a meta-analysis. Biogeosciences 12, 1671–1682. (10.5194/bg-12-1671-2015)

[9] Bach LT, Riebesell U, Gutowska MA, Federwisch L, Schulz KG 2015 A unifying concept of coccolithophore sensitivity to changing carbonate chemistry embedded in an ecological framework. Prog. Oceanogr. 135, 125–138. (10.1016/j.pocean.2015.04.012)

[10] Young JR 1994 Functions of coccoliths. In Coccolithophores (eds Winter A, Siesser WG), pp. 63–82. Cambridge, UK: Cambridge University Press.

[11] Flynn KJ, Blackford JC, Baird ME, Raven JA, Clark DR, Beardall J, Brownlee C, Fabian H, Wheeler GL 2012 Changes in pH at the exterior surface of plankton with ocean acidification. Nat. Clim. Change 2, 510–513. (10.1038/nclimate1696)

[12] Flynn KJ et al. 2015 Ocean acidification with (de)eutrophication will alter future phytoplankton growth and succession. Proc. R. Soc. B 282, 20142604 (10.1098/rspb.2014.2604) PMC4375859 25716793

[13] Taylor AR, Russell MA, Harper GM, Collins TFT, Brownlee C 2007 Dynamics of formation and secretion of heterococcoliths by *Coccolithus pelagicus* ssp *braarudii*. Eur. J. Phycol. 42, 125–136. (10.1080/09670260601159346)

[14] Paasche E 2001 A review of the coccolithophorid *Emiliania huxleyi* (Prymnesiophyceae), with particular reference to growth, coccolith formation, and calcification–photosynthesis interactions. Phycologia 40, 503–529. (10.2216/i0031-8884-40-6-503.1)

[15] Brownlee C, Taylor AR 2004 Calcification in coccolithophores: a cellular perspective. In Coccolithophores: from molecular processes to global impact (eds Thierstein HR, Young JR), pp. 31–49. Berlin, Germany: Springer.

[16] Herfort L, Loste E, Meldrum F, Thake B 2004 Structural and physiological effects of calcium and magnesium in *Emiliania huxleyi* (Lohmann) Hay and Mohler. J. Struct. Biol. 148, 307–314. (10.1016/j.jsb.2004.07.005) 15522779

[17] Trimborn S, Langer G, Rost B 2007 Effect of varying calcium concentrations and light intensities on calcification and photosynthesis in *Emiliania huxleyi*. Limnol. Oceanogr. 52, 2285–2293. (10.4319/lo.2007.52.5.2285)

[18] Leonardos N, Read B, Thake B, Young JR 2009 No mechanistic dependence of photosynthesis on calcification in the coccolithophorid *Emiliania huxleyi* (Haptophyta). J. Phycol. 45, 1046–1051. (10.1111/j.1529-8817.2009.00726.x) 27032349

[19] Bach LT, Mackinder LCM, Schulz KG, Wheeler G, Schroeder DC, Brownlee C, Riebesell U 2013 Dissecting the impact of CO_2_ and pH on the mechanisms of photosynthesis and calcification in the coccolithophore *Emiliania huxleyi*. New Phytol. 199, 121–134. (10.1111/nph.12225) 23496417

[20] Hansen PJ 2002 The effect of high pH on the growth and survival of marine phytoplankton: implications for species succession. Aquat. Microbial Ecol. 28, 279–288. (10.3354/ame028279)

[21] Clark DR, Flynn KJ, Fabian H 2014 Variation in elemental stoichiometry of the marine diatom *Thalassiosira weissflogii* (Bacillariophyceae) in response to combined nutrient stress and changes in carbonate chemistry. J. Phycol. 50, 640–651. (10.1111/jpy.12208) 26988448

[22] Page S, Hipkin CR, Flynn KJ 1999 Interactions between nitrate and ammonium in *Emiliania huxleyi*. J. Exp. Mar. Biol. Ecol. 236, 307–319. (10.1016/S0022-0981(98)00212-3)

[23] Schlüter L, Lohbeck KT, Gutowska MA, Gröger JP, Riebesell U, Reusch TBH 2014 Adaptation of a globally important coccolithophore to ocean warming and acidification. Nat. Clim. Change 4, 1024–1030. (10.1038/nclimate2379)

[24] Bach LT, Riebesell U, Schulz KG 2011 Distinguishing between the effects of ocean acidification and ocean carbonation in the coccolithophore *Emiliania huxleyi*. Limnol. Oceanogr. 56, 2040–2050. (10.4319/lo.2011.56.6.2040)

[25] Suffrian K, Schulz KG, Gutowska MA, Riebesell U, Bleich M 2011 Cellular pH measurements in *Emiliania huxleyi* reveal pronounced membrane proton permeability. New Phytol. 190, 595–608. (10.1111/j.1469-8137.2010.03633.x) 21294736

[26] Taylor AR, Chrachri A, Wheeler G, Goddard H, Brownlee C 2011 A voltage-gated H^+^ channel underlying pH homeostasis in calcifying coccolithophores. PLoS Biol. 9, e1001085 (10.1371/journal.pbio.1001085) 21713028 PMC3119654

[27] Tyrrell T, Merico A 2004 *Emiliania huxleyi*: bloom observations and the conditions that induce them. In Coccolithophores: from molecular processes to global impact (eds Thierstein HR, Young JR), pp. 75–97. Heidelberg, Germany: Springer.

[28] Daniels CJ, Sheward RM, Poulton AJ 2014 Biogeochemical implications of comparative growth rates of *Emiliania huxleyi* and *Coccolithus* species. Biogeosciences 11, 6915–6925. (10.5194/bg-11-6915-2014)

[29] Mackinder L, Wheeler G, Schroeder D, Riebesell U, Brownlee C 2010 Molecular mechanisms underlying calcification in coccolithophores. Geomicrobiol. J. 27, 585–595. (10.1080/01490451003703014)

[30] Marsh ME 2003 Regulation of CaCO_3_ formation in coccolithophores. Comp. Biochem. Physiol. B 136, 743–754. (10.1016/S1096-4959(03)00180-5) 14662299

