# Supplementary material for: The role of coccolithophore calcification in bioengineering their environment
Source: Proc Biol Sci. 2016 Jun 29;283(1833):20161099. doi: 10.1098/rspb.2016.1099 (PMC4936047; doi:10.1098/rspb.2016.1099)
Supplement: Additional Materials and Methods [file rspb20161099supp1.docx]

**The role of coccolithophore calcification in bioengineering their environment**

Kevin J. Flynn, Darren R. Clark, Glen Wheeler

Supplementary Materials

Materials and Methods

*Base model of phytoplankton growth and carbonate chemistry*

Modelling was undertaken using the same platform (Powersim Constructor v2.51, Isdalstø, Norway) and source model code as deployed previously [11]. This included a variable stoichiometric acclimative model of phytoplankton physiology, coupled to a carbonate chemistry module.

The model of algal physiology provided a variable stoichiometric (i.e., C:N:P:Chl) description of phytoplankton growth, including limitations by resource availability (CO_2_, nitrate, ammonium, phosphate, light). The model structure has been developed and deployed as a dynamic description for many phytoplankton over the last 15+ years, most recently and including for *Emiliania* as in [12].

The carbonate chemistry component of the model (used also in [11,12] and in [31]) yields values for the carbonate chemistry system, interrelating pH, total alkalinity (TA) and DIC, that are very similar to those generated by the CO2sys software [32]. Here, these values (pH, TA, DIC and thence the contributions of DIC between H_2_CO_3_, HCO_3_^-^ and CO_3_^- -^) were computed during the simulations based on the initial start parameters for pH, TA, DIC, and nutrients. The closeness of model fits to experimental data, and of the emergent pH values from the model versus pH values logged during experiments, as we have shown previously [12], provides confidence in the computed values of TA and DIC and thence that the platform we have used for this work is sound.

The model reports bulk water [H+] and also that proximal to the cells. As we are considering cells of only ca. 5µm diameter, and hence diffusion rapidly establishes homogeneity, these values are for practical purposes identical [11].

Our hypothesis is that, as growth at a stable pH aids growth [12,21], *Emiliania* would optimise growth by modifying the ratio of PIC:POC production (PIC:POC_prod_). To explore the impacts of growing at different PIC:POC_prod_ upon the stability of pH (of [H^+^]) we removed the previously configured relationship between pH and growth rate [12]. We assumed for each simulation a fixed ratio of calcification set against C-fixation (i.e., set PIC:POC_prod_), computing changes in the carbonate chemistry accordingly, as we have before [11,12].

*Simulations of open water pH scenarios*

Simulations were run within scenarios of a water column with gas exchange at the surface, and exchange of water at the interface of the mixed layer. The basis of these simulations in terms of abiotic factors is the same as used previously [11,12], though lacking simulated predator activity. Growth was simulated in various water column scenarios, similar to those used before [11,12]; surface light was provided at 1000µmol m^-2^ s^-1^ in a 12:12 L:D cycle, and nutrients were supplied at a default 16 µM nitrate-N, 1 µM phosphate-P. At the peak of growth, just prior to N-exhaustion, this would support development of a bloom of 5 µm diameter cells with a mass ratio of C:N of 6 and a cell-C density of 200gC (cell-L)^-1^ [25,33,34], in the region of 80 x 10^6^ cells L^-1^ from a start of ca. 0.6 x 10^6^ cells L^-1^.

*Calculation of δ[H^+^]*

Simulations were run for either a simulated 20 or (for slow µ_max_) 40 days, until cell growth had halted due to cellular N:C having fallen to close to its minimum value (NC_min_). Effectively this simulates bloom growth through to its N-limited conclusion. Over the period of the simulated growth, model output included changes in [H^+^], varying over the diel light-dark cycle. The extreme minimum and maximum values of [H^+^] (i.e., [H^+^]_min_ and [H^+^]_max_, respectively) normalized against the initial (atmosphere-equilibrated pCO_2_) value of [H^+^] (i.e., [H^+^]_init_) were used to calculate δ[H^+^].

Thus, δ[H^+^], displayed as %, is given by the following -

$$\boldsymbol{\delta}\left[ \boldsymbol{H}^{\boldsymbol{+}} \right]\boldsymbol{=}\frac{\left( {\boldsymbol{[}\boldsymbol{H}^{\boldsymbol{+}}\boldsymbol{]}}_{\boldsymbol{max}}\boldsymbol{+}{\boldsymbol{[}\boldsymbol{H}^{\boldsymbol{+}}\boldsymbol{]}}_{\boldsymbol{min}}\boldsymbol{-2\cdot}{\boldsymbol{[}\boldsymbol{H}^{\boldsymbol{+}}\boldsymbol{]}}_{\boldsymbol{init}} \right)}{{\boldsymbol{[}\boldsymbol{H}^{\boldsymbol{+}}\boldsymbol{]}}_{\boldsymbol{init}}}\boldsymbol{\cdot100}$$

This approach was used, rather than considering average differences between [H+] and [H^+^]_init_ over the whole bloom growth period, because for an organism [H^+^] (i.e., pH) need only transit to (sub-) lethal levels for a short period for damage to be caused. Thus, the critical issue for an organism is the extreme values (high and/or low) of [H^+^] encountered during growth.

**References in addition to those in the main text**

31. Artioli Y, Blackford JC, Nondal G, Bellerby RGJ, Wakelin SL, Holt JT, Butenschön M, Allen JI. 2013. Heterogeneity of impacts of high CO_2_ on the North Western European Shelf. *Biogeosci*. **10**, 9389-9413.

32. Pierrot D, Lewis E, Wallace DWR. 2006. CO2sys MS Excel Program Developed for CO_2_ System Calculations. ORNL/CDIAC-105. Carbon Dioxide Information Analysis Center, Oak Ridge National Laboratory, US Department of Energy, Oak Ridge, TN, U.S.A.

33. Flynn KJ, Page S, Wood G, Hipkin CR. 1999. Variations in the maximum transport rates for ammonium and nitrate in the prymnesiophyte *Emiliania huxleyi* and the raphidophyte *Heterosigma carterae*. *J. Plankt. Res.* **21**; 355-371.

34. Menden Deuer S, Lassard EJ. 2000. Carbon to volume relationships for dinoflagellates, diatoms, and other protist plankton. *Limnol. Oceanogr.*, **45**, 569–579
